# The application of Box-Behnken-Design in the optimization of HPLC separation of fluoroquinolones

**DOI:** 10.1038/s41598-019-55761-z

**Published:** 2019-12-19

**Authors:** Andrzej Czyrski, Justyna Sznura

**Affiliations:** Poznań University of Medical Sciences, Department of Physical Pharmacy and Pharmacokinetics, Święcickiego 6 Street, 60-781 Poznań, Poland

**Keywords:** Drug development, Preclinical research

## Abstract

Box-Behnken Design is a useful tool for the optimization of the chromatographic analysis. The goal of this study was to select the most significant factors that influenced the following parameters of the chromatographic separation: retention time, relative retention time, symmetry of the peaks, tailing factor, a number of theoretical plates, Foley – Dorsey parameter, resolution factor, peak width at half height. The results underwent the ANOVA test to find the statistically significant variables and interactions between them. The level of significance was for *p* < *0.05*. The polynomial equations described quantitatively the statistically significant parameters and the interactions between them. The statistical analysis indicated both the best conditions for the separation of the compounds and the variables that were most influential for peaks’ parameters. The four-factor analysis performed for LEVO and MOXI indicated that ACN, TEA and pH are the most significant factors that influence the separation. The analysis for the pair CIPRO and LEVO required six factors. The statistical analysis proved that the most significant factors are ACN, MeOH and TEA. In the separation of these two compounds on the HPLC column, the interaction ACN × MeOH was also significant.

## Introduction

Fluoroquinolones are the antibacterial drugs that possess a broad bactericidal activity against Gram-positive, Gram-negative and atypical bacteria. Their activity strongly depends on their concentration. To provide an effective therapeutic drug monitoring, it is necessary to develop a method which is fast, reproducible and reliable. The development of the new method involves the optimization of the separation conditions. It can be time-consuming and resource-demanding due to the large number of the settings such as the content and composition of an organic phase, the acidity of the mobile phase, the presence of additives (ion-pair reagent or salts), the flow of the mobile phase. They should be considered when the optimal conditions of chromatographic separation are to be found. Design of experiment is a useful methodology which examines the influence of the independent variables on chromatographic separation parameters. It also indicates the interactions between the parameters which is not possible in the one-factor analysis. The designs such as Doehlert Design, Box-Behnken Design (BBD) or Central-Composite Design can be applied to optimize the chromatographic conditions. The application of a proper design leads to the save of the time and also considers the interactions between the factors. It results in satisfactory separation and satisfactory values of the dependent variables^1–5^.

The traditional univariate procedure is not satisfactory because it involves the level change only in one factor while the others remain unchanged^6^. On the other hand, the full factorial design comprised many experiments. In 1960 Box and Behnken proposed the three factorial analysis combined in blocks^7^. The main advantage of this analysis was the reduction of the number of experiments. BBD does not contain the combinations in which all variables are on the highest or the lowest levels. Testing the values at such levels may lead to unsatisfactory results. On the other hand, this design is also not indicated when we would like to know the response at the extreme values of independent variables^8^.

BBD can be applied in the chromatographic analysis in the optimization of chromatographic procedure^9–11^. The ANOVA analysis indicated both the most significant factors in the separation of fluoroquinolones and revealed many mutual interactions. They were observed for the factors which were not singnificant when analysed in single. It revealed the complexity of the chromatographic separation of fluoroquinolones, which was the result of the zwitterionic nature of these drugs. The following responses will be analysed in this study: retention time, relative retention time, tailing factor, the symmetry of the peaks, peak width Foley-Dorsey parameter, resolution factor, the number of theoretical plates. In our study, we applied BBD for the six-factor analysis in chromatographic separation of levofloxacin (pKa_1_ 6.05) and ciprofloxacin (pKa_1_ 6.09) as an example of the pair compounds with similar pK_a_ and lipophicity which make them migrate simultaneously. Four-factor analysis was performed for the compounds that have different pK_a_ – levofloxacin (pKa_1_ 6.05) and moxifloxacin (pKa_1_ 6.4)^12^. BBD is not commonly used in investigating difficult chromatographic separations with a relatively large number of responses, and therefore this paper aims to assess the usefulness of BBD for such an application.

## Results and Discussion

In BBD analysis in many studies were there considered designs for three or four independent variables. In our study, we started with three factors. In CIPRO and LEVO case it was not possible to separate the analytes – the peaks overlapped. It was necessary to introduce extra independent variables to get the satisfactory separation. For MOXI and LEVO the separation was sufficient, but the R^2^ adjusted was not sufficient. The introduction of the fourth factor in LEVO/MOXI analysis and the extension the number of independent variables to six in LEVO/CIPRO analysis made it possible find polynomial equations that could describe the designs properly. All the designs were significant and well fitted - the R^2^ was close to one and the p-value was below 0.0001 (Supplementary Tables S1–S2). The lack of fit was not significant. The polynomial equations that describe the significant factors and the interactions between them are listed in the Part B of the Supplementary Information.

### The optimization of the chromatographic separation

The assumptions we adopted in our study are listed below:

- The use of the organic solvents should be as low as possible.
- The applied pH should not be below 2.0.
- The retention time for the first analyte should be at least 2.5 min and it should not exceed 10 min.
- The symmetry factor and tailing factor should not exceed 1.5.
- The number of theoretical plates and Foley-Dorsey parameter should take the highest values.
- The R_s_ factor should be at least 2.0.
- The peak width in half height should be as minimum as possible.

### Retention time and relative retention time

According to six-factors design, the significant factors that influence retention time of LEVO and CIPRO are acetonitrile (ACN) and methanol (MeOH) content, flow, the interactions between ACN and MeOH, ACN and flow and ACN^2^. For these variables the *F-values* in the ANOVA test are the highest. However retention time for CIPRO is more sensitive than LEVO to the change of the content of the organic solvent for both ACN and MeOH. The increase of MeOH content resulted in both the prolongation of the retention time and not symmetric shape of the peaks. On the other hand too much ACN in the mobile phase caused the overlapping of the peaks. The decrease of the ACN content for both analytes would result in a significant increase of the retention time. However, it is more significant for CIPRO than for LEVO (higher values of *F* in ANOVA analysis and coefficients in the equations). The following proportion in the mobile phase between ACN and MeOH - 12:17 is optimal. The proper resolution of the peaks and the symmetry was also achieved. Further increase of both contents in the mobile phase would not significantly speed up the retention time for both compounds. It would result only in producing wastes with high organic content. The statistical analysis also proved that the mutual interaction between ACN and MeOH is the most significant one (Supplementary Table S3a).

Retention time might be influenced by the changes in the pressure during the analysis. To reduce the impact of the external variables, the RRT might also be applied in the HPLC analysis. The most significant factors that influenced the RRT for both analytes are similar for those that influence retention time (Supplementary Table S3b).

The four-factor analysis for MOXI and LEVO separation proved that ACN content have a predominant impact on retention time of LEVO and MOXI (Supplementary Table S4a). The ACN concentration 25–28% would result in a satisfactory short retention time for both analytes. The further increase in the ACN concentration could result in the overlapping of the peaks for both analytes. In the case of RRT for both analytes, the significant factors were ACN, TEA, and ACN^2^. The content of ACN was a predominant factor (Supplementary Table S4b).

### Peak shape (symmetry, tailing factor and width of the peaks)

The parameters that characterize the shape of the peaks are the symmetry, the tailing factor and the peak width in half height. Fluoroquinolones are the compounds for which the asymmetry and tailing of the peaks are observed during the chromatographic analysis. It is caused by the zwitterionic structure – they possess carboxyl group that can be negatively charged and the nitrogen atoms that can be positively charged. Fluoroquinolones are charged within all pH range. The optimal values for symmetry of the peaks are 0.9–1.2 and for tailing is 1.2–1.5. These values are often hard to reach because of strong ionic interactions with the stationary phase. The acceptable value is up to 1.5. The tailing of the peaks is caused by the hydrophobic and ionic interactions with the silanol groups of the stationary phase. The use of ion-pair reagent combined with a proper pH reduces both the availability of free stationary phase silanols and the analyte’s interaction with them. The addition of ion pair reagent should be as low as possible. The high content might cause a long column equilibrating time, and it is difficult to wash off the column^13–15^.

The six-factor analysis proved that the symmetry of the CIPRO peak is mainly influenced by pH and the interaction MeOH × TEA (trimethylamine) (Supplementary Table S3c). The impact of pH, quadratic term of flow and TEA^2^ on the shape is more significant than TEA in single. The other significant factors are: TEA × NaH_2_PO_4_ and pH^2^. The analysis of the RSM diagram proved, that the decrease in pH and increase in TEA concentration make the peaks symmetric. The observed lowest value for the symmetry is observed for pH 1.8–3.0 and for TEA concentration 0.8–1.2%. The increase of pH results in lack of symmetry of the peaks. It is noted for pH above 3.6. Even high concentration of TEA will not improve the shape of the peaks in this case (Supplementary Fig. S1a). For LEVO, the most significant factor is ACN and its interaction with TEA. The other very important interactions are MeOH × TEA, MeOH^2^, TEA^2^, pH^2^. The increase in ACN and decrease in MeOH level leads to the increase of the symmetry factor. The lowest values for symmetry factor might be observed for pH below 2.0 and the TEA content 0.7–1.5%. The increase in pH to the value 2.6 for the previously mentioned range of TEA concentration resulted in symmentry factor which did not exceed the value 1.2 (Supplementary Fig. S1b). As well as for CIPRO the high pH combined with the low content of TEA resulted in lack of symmetry for peaks. The optimum concentration of TEA occurred to be 0.7% and pH 2.5 for both analytes. For the TEA concentration below 0.8%, the symmetry factor approaches the value 1.0–1.2. The impact of pH on peak symmetry was more significant in the case of CIPRO than LEVO. The very low pH is not applicable due to the restricted column capacity – the pH below 2.0 can damage the column. The application of pH 2.5 resulted in a satisfactory symmetry factor of the peaks.

In the four-factor analysis, the critical factors for LEVO peak’s symmetry are ACN and pH^2^ (Supplementary Table S4c). The following interactions ACN × pH and NaH_2_PO_4_ × pH also have an impact on the signal's shape. According to the RSM diagram, the increase in ACN concentration leads to higher values of the symmetry factor. However the maximum is reached at 25–26%, and then it decreases. The shape of the RSM diagram indicates that the satisfactory symmetry level within the following ranges is for the concentration of the phosphate buffer 20–40 mM and for pH 2.4–3.4 (Supplementary Fig. S2a). The concentration of the phosphate buffer when analysed with the ACN content does not affect the studied system. For MOXI symmetry the most influential factors were pH, TEA, ACN and TEA × pH (Supplementary Table S4c). The increase in pH and decrease in TEA concentration results in the lack of peak symmetry as well as for the remain analytes (Supplementary Fig. S2b). The impact of TEA on symmetry is significant when the concentration of ACN increases. The optimum value for symmetry was at pH 2.0–2.5 and ACN content within a range 18–28%. This concentration range combined with a TEA concentration 1.4–1.5% provides the optimum value for symmetry. The further increase in ACN and decrease in TEA concentration make signals not symmetric and tailing.

The tailing factor of CIPRO peaks is influenced mainly by TEA, pH, flow^2^, TEA^2^, TEA × pH, MeOH and ACN (Supplementary Table S3d). The change of ACN content has an impact on tailing factor of CIPRO at higher values of pH. The lowest tailing of the peaks might be observed for low pH - the optimum value of tailing factor can be observed for ACN concentration 6–14%. The application of the lower ACN content could retain the tailing factor on the satisfactory level, however it could result in the longer retention time of both CIPRO and LEVO. The peaks might be symmetric but broad (Supplementary Fig. S3a). The lowest content of MeOH that reduces the peaks tailing is 17% for MeOH. The more MeOH is added, the lower tailing factor is observed. However to reduce the use of the organic solvents, the 17% content of MeOH was found appropriate. The optimum values of tailing factor for CIPRO are observed for pH 2.0–3.5. The lower values of pH combined with low content of TEA (0.6%-0.8%) resulted in satisfactory level of tailing factor. For higher pH (up to 3.8) it is possible to reduce the tailing of the peaks, but it requires the higher concentrations of TEA. The tailing is also reduced in the phosphate concentrations range 45–50 mM. The optimal value of tailing factor is noted for TEA concentration 0.6–1.4%, pH 2.0–2.5 and NaH_2_PO_4_ concentration 45–50 mM.

The statistical analysis proved that for LEVO in the six-factor analysis the most significant factor that influences the tailing of the peaks is ACN (Supplementary Table S3d). The other parameters are MeOH, ACN × TEA, MeOH × TEA, TEA^2^, pH^2^. The RSM graph shows that the increase of ACN and MeOH concentration reduces the tailing of LEVO peaks. The tailing factor 1.2–1.5 was observed for the following concentration ranges: 10–13% for ACN and 16–18% for MeOH. The decrease of the ACN concentration resulted in the increase of tailing factor (Supplementary Fig. S3b). The higher values of pH and a decrease in TEA concentration causes the tailing of the LEVO peaks. The range of pH and TEA concentrations in which the tailing of the peaks is reduced is broad. It comprises the range 0.7%-1.5% for TEA and pH 2.0–4.0. However the higher values of pH could have also an impact on symmetry. The optimal tailing factor values are observed for TEA 0.6–1.2% and pH 2.4–2.8. The concentration of the phosphate buffer should be ca. 50 mM.

The four-factor analysis indicates that the crucial factors for tailing of MOXI peaks are ACN, pH and TEA. The impact of pH on tailing is significant at its higher values (Supplementary Fig. S4a). The tailing factor is reduced at the lower content of ACN and growing concentration of TEA. The increase of TEA concentration and a decrease in pH reduces the tailing of the MOXI peaks. The optimum values of MOXI tailing are observed for pH 2.4-2.8, the ACN 26–28% and TEA 1.4–1.5%. The four-factor analysis for LEVO indicated the same significant parameters as for MOXI and also the additional interactions ACN × TEA, NaH_2_PO_4_^2^ and pH^2^ (Supplementary Table S4d). The maximum tailing is observed at the content of ACN 24–30% and pH range 4.0–4.2. The lowering of the pH level caused the reduction of the tailing of the peaks and it is observed within the following pH range 2.2–3.2 (Supplementary Fig. S4b). The tailing of the LEVO peaks is also reduced at the concentration of the phosphate buffer 20–40 mM and high TEA concentrations. The most optimal values are observed for NaH_2_PO_4_ concentration 30–35 mM, pH 2.2–2.5 and TEA concentration 1.0–1.5%.

The six-factor analysis proved that the most significant factors that influenced the peak width in half height of CIPRO and LEVO were ACN, MeOH and flow (Supplementary Table S3e). The interaction ACN × MeOH was also significant. The peak width in half height decreases as the percentage content of MeOH and ACN increases. The satisfactory peak width in half height is observed for ACN and MeOH concentrations ranges 12–14% and 16–19% respectively (Supplementary Fig. S5). The observed value is ca. 0.2 min. The high content of organic solvent would also result in the low value of peak width in half height. However the application of high concentration of both organic solvents is not possible because it would cause the overlapping of the peaks. The lowest peak width in half height was noted for the flow 1.0–1.2 ml/min. For the low content of ACN or MeOH, the peak width broadens with the increase of pH. It reaches the maximum at pH 3.2 and it starts to narrow. With the increasing content of organic solvents the impact of pH, TEA and NaH_2_PO_4_ on peak width in half height ceases for CIPRO and LEVO. The optimal pH condition were 2.5 and high NaH_2_PO_4_ concentration. In the four-factor analysis, the peak width of LEVO and MOXI was influenced by ACN, pH, and ACN^2^ (Supplementary Table S4e). However ACN in single influences much stronger the peak width in half height of MOXI than LEVO. For LEVO more significant is the quadratic term of ACN. According to RSM diagram, the lowest value for peak width was observed with the increasing content of ACN. It reaches the minimum at the content 26–32% for LEVO and 26–36% for MOXI and for the pH range 2.0–2.6 for both analytes (Supplementary Fig. S6). For MOXI the concentration of NaH_2_PO_4_ is not a significant factor when analysed in single, contrary to its interaction with ACN. The minimum value of peak width in half height is observed for NaH_2_PO_4_ concentration 30–34 mM for LEVO, and 33–38 mM for MOXI. The combination with the ACN content 27% and 1.5% of TEA resulted in low peak width in half height. It was also confirmed during the preliminary study - we needed to introduce the fourth factor to LEVO/MOXI analysis. It was NaH_2_PO_4_ concentration. Without it we could not find a proper design, that describes the values completely. The introduction of an extra variable resulted in improving the value of R^2^ adjusted. For MOXI the maximum peak width is observed for 0.8–1.2% TEA and 2.6–3.6 pH. In case of LEVO, the maximum peak width was observed for 0.7–1.1%TEA and the pH 3.8–4.2. The peak width at half height is a parameter that is associated with the retention of the analyte in the HPLC system. It is a result of an axial diffusion of the analyte on the column. The parameters that are statistically significant for retention of the analytes are also significant for peak width in half height. In the abovementioned correlations for CIPRO, LEVO and MOXI the key role played pH and TEA. The high tailing is caused by the negatively charged silanol groups from the stationary phase and the positively charged amine group of the fluoroquinolone. The decrease of pH value to 3.5 reduces it. The silanol groups above pH 3.5 are ionized and interact with 1° and 2° amines, and this is the reason why the high tailing factor values are observed for pH 4.5 and 5.5^10^. The proper TEA concentration combined with acidic pH decreases the second order ionic interactions between the ionized carboxyl and amine groups of the analytes. The acidic pH suppresses the ionization of carboxyl group, and TEA as an ion pair reagent blocks the access to silanol groups in the stationary phase. For analyzed compounds, the acidic pH reverses the ionization of the carboxyl group, and the addition of TEA blocks the silanol groups is a stationary phase which resulted in a reduction of the tailing of the peaks. The application of acidic pH, the proper buffer concentration or ion pair reagent is essential for reducing the tailing of the peaks and improving the symmetry^6,16^.

### The number of theoretical plates, Foley-Dorsey parameter and R_s_

The ANOVA test in the six-factor design for LEVO analysis proved that the factors which are the most significant for the number of theoretical plates are as follows ACN, TEA, ACN × TEA and pH (Supplementary Table S3f). The statistically significant are also TEA × NaH_2_PO_4_ and NaH_2_PO_4_^2^. The increase of ACN content and decrease of pH result in an increase in the number of theoretical plates. The impact of the concentration of TEA on the number of theoretical plates is significant at the ACN content of at least 7%. At the pH 2.5 (and lower) and the ACN concentration 12%, the capacity of the column reached the maximum (Supplementary Fig. S7a). The maximum value of the number of theoretical plates is observed for TEA range 0.7–1.4%. The low value of pH combined with the concentration of 40–50 mM NaH_2_PO_4_ results in the increase in the number of theoretical plates. The increase in the flow leads to the increase in the number of theoretical plates. At 1 ml/min it reaches the maximum, then it decreases. The four-factor analysis for LEVO indicated ACN, pH and ACN^2^ as the most influential factors (Supplementary Table S4f). The growth of the number of theoretical plates is not linear for ACN, because too high or too low concentration results in the decreasing number of theoretical plates for LEVO. The RSM diagram indicates that the highest number of theoretical plates for LEVO is for the content of ACN 24–32% and it increases with the decrease in pH (Supplementary Fig. S8a).

In the CIPRO case, the factor that influenced most the capacity of the column was MeOH, flow and TEA × NaH_2_PO_4_. The impact of TEA on the number of theoretical plates for CIPRO is significant for low concentration of MeOH. Contrary to LEVO, the TEA considered as a single factor is not statistically significant. On the other hand its mutual interaction with the concentration of the phosphate buffer has a similar impact to column capacity as MeOH – the *F-values* are similar (Supplementary Table S3f). The interaction of TEA with ACN was also significant. The maximum number of theoretical plates for CIPRO is observed for ACN concentration at least 12% and TEA 0.7–1.2%. The highest number of theoretical plates for CIPRO is reached for the NaH_2_PO_4_ concentration 40–50 mM and pH 2.0–2.5. The RSM diagram of the the number of theoretical plates for CIPRO versus MeOH and pH has four maxima. The lowering of the content of MeOH in the mobile phase boosts the column capacity. The maximum value of the number of the theoretical plates was reached for the MeOH concentration of 16–17% and pH 2.4–3.2 (Supplementary Fig. S7b).

The factors that influenced most the number of theoretical plates of MOXI were the ACN, pH and ACN^2^ (Supplementary Table S4f). The RSM diagram indicated one maximum which is observed at ACN concentration range 22–28% and pH 2.0–2.8%. The further increase in both ACN and pH resulted in the lowering of the number of theoretical plates (Supplementary Fig. S8b).

The fluoroquinolones peaks are not prone to take a classical Gaussian shape. In this case the capacity of the column may be described also by the Foley-Dorsey parameter. It might be considered for evaluation of the capacity of the column when the peaks are not symmetric.

The analysis of the Foley-Dorsey in six-factor design proved that for LEVO the most significant factors are ACN, ACN^2^ and ACN × MeOH (Supplementary Table S3g). The increase in ACN and MeOH causes the rise of Foley-Dorsey (Supplementary Fig. S9a). The impact of pH and TEA are not as significant as in the case of a number of theoretical plates. The ANOVA test proves that they are not significant in single, but their interactions such as: ACN × pH, MeOH × pH, TEA × pH, TEA × flow, TEA^2^ and pH^2^ are significant. The increase of ACN concentration results in a better capacity of the column for LEVO regardless the used pH. However the highest values of Foley-Dorsey for LEVO are noted for the low TEA concentrations (0.6–0.8%) at pH 2.0–2.5. The maximum values of Foley-Dorsey for LEVO are registered for the flow 1 ml/min.

In case of CIPRO, the most significant is MeOH content and the ACN × MeOH, pH and flow. The concentration of phosphates was not statistically significant, contrary to its quadratic term. The RSM diagram for Foley-Dorsey versus MeOH and pH (Supplementary Fig. S9b) is similar to the RSM diagram for the number of theoretical plates for CIPRO (Supplementary Fig. S7b). It also possesses four maxima. The highest Foley-Dorsey value is observed for MeOH 16–18% and pH 2.2–3.6.The high concentration of phosphates (ca. 50 mM) combined with the pH 2.2–2.5 and ACN concentration of 12% resulted in high value of Foley-Dorsey for CIPRO.

In the four-factor analysis, the factors that are crucial for the Foley-Dorsey for MOXI are TEA, pH and the ACN × pH and pH^2^. For LEVO the most significant factors are pH and ACN^2^ (Supplementary Table S4g). The higher concentration of TEA and the lower pH make the Foley-Dorsey values for MOXI and LEVO increase (Supplementary Fig. S10). However too much ACN would resulted in peaks’ overlapping. The pH 2.5 combined with the concentration of ACN 25–27% and the 33 mM NaH_2_PO_4_ concentration resulted in high value of Foley-Dorsey for both analytes.

The parameter that describes the resolution of the analytes is R_s_. The value of R_s_ at least 1.5 means that the peaks are fully resolved. This value is reliable for the symmetric peaks. In case of asymmetry, the minimum value is at least 1.7–2.0. We assumed that the goal value should be at least 2.0 and higher. The separation of LEVO and CIPRO on the column occurred to be difficult - they are eluted at the same time. According to ANOVA test, the most significant factors were ACN, MeOH and pH. TEA and NaH_2_PO_4_ were also significant however the highest *F-values* are observed for the former three factors (Supplementary Table S3h). The interaction ACN × MeOH was significant for the resolution of the analytes. The use of MeOH without ACN resulted in long retention time and the peaks were broad. On the other hand, the use of ACN without MeOH resulted in overlapping the peaks. The critical factor in the separation of these two peaks was a specific ratio of MeOH and ACN in the mobile phase. MeOH makes the peaks separate, ACN makes them elute in a reasonable time. TEA, phosphates and proper value of pH make a good shape, reduce the tailing, and improve the capacity of the column. The significant interaction for resolution of the peaks are ACN × TEA, ACN × pH, ACN^2^ and TEA^2^. The decrease of MeOH concentration and increase in TEA result in increasing the R_s_ factor. The satisfactory value was achieved for 17% for MeOH and at least 0.7% for TEA. The further increase in TEA concentration may lead to better resolution, however it was not significant growth (Supplementary Fig. S11a). These two parameters combined with a 12% content for ACN, pH 2.5 and 50 mM NaH_2_PO_4_ concentration resulted in value of Rs above 2.0. The lower content of organic solvents result in broad and tailing peaks.

The key factor influencing the resolution between LEVO and MOXI was ACN. The ANOVA test indicates also its quadratic term (ACN^2^) as an important factor (Supplementary Table S4h). The others are pH and its interaction with ACN. The decrease in ACN content increases the value of R_s_ (Supplementary Fig. S11b). The low content of ACN results in broad peaks and significant tailing and long time of analysis. Both pH and TEA have limited impact on the R_s_ for the pair LEVO/MOXI when considered with ACN. The combination of pH 2.5 and NaH_2_PO_4_ concentration of 50 mM resulted in Rs above 7.0.

For both pair of analyzed compounds ACN, pH and pH^2^ were the significant factors for a total resolution of the peaks. Szerkus *et al*. and Kasagić *et al*. also indicated the pH as a statistically significant factor in HPLC separation^6,17^.

### The most optimal conditions for separation of the investigated compounds

**Figure 1:**
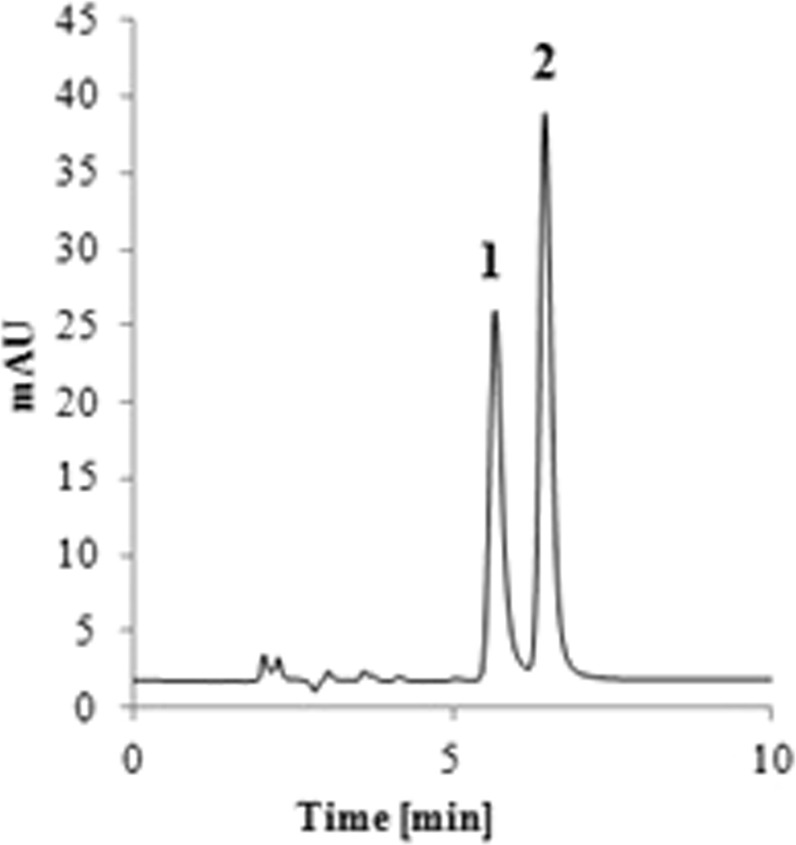
The chromatogram of LEVO (1) and CIPRO (2) for optimized conditions.

**Figure 2:**
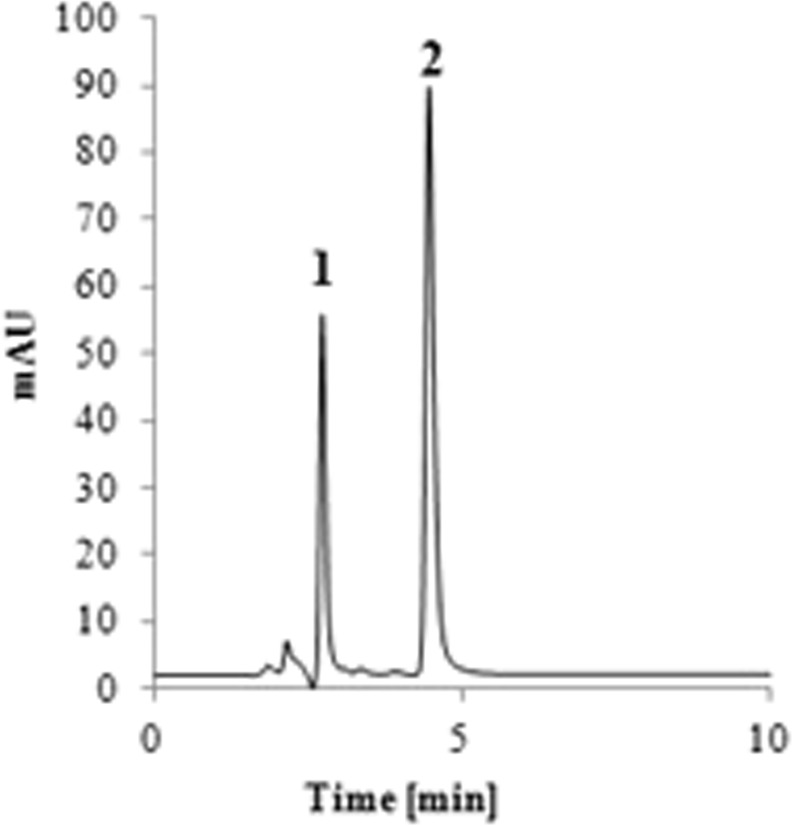
The chromatogram of LEVO (1) and MOXI (2) for optimized conditions.

The aim of the optimization of the chromatographic separation was to find such conditions that provide the best values of the analysed factors. The conducted analysis proved that the independent variables can interact with one another in many ways. The other point, which was also considered, was the rational use of the reagents – it is very important from the ecological and economical point of view. The most optimal conditions for the separation of LEVO and CIPRO were as follows: ACN: MeOH: 0.7% TEA: 50 mM NaH_2_PO_4_ (12:17:35.5:35.5), the pH of the mobile phase was 2.5 and the flow was 1 ml/min. The chromatogram is presented in the Fig. 1. The retention time of LEVO and CIPRO was 5.41 min and 6.19 min., respectively. Under these conditions the use of the organic solvents is reduced. It was necessary to use ACN at 12% level because it speeded up the time of analysis and also have beneficial impact on other parameters. It resulted in the reduced use of MeOH which was crucial for separation of the peaks of the analytes. The applied pH is 2.5. The symmetry and tailing factor for analytes were satisfactory within the pH ranges 2.0–2.5. To avoid the pH at the extreme value of 2.0 we decided to apply the pH of 2.5. The capacity of the column reached its maximum at the flow 1.0–1.2 ml/min. To avoid excessive use of the mobile phase the flow was set on 1.0 ml/min. NaH_2_PO_4_ is on its maximum value. It is caused by the fact that LEVO and CIPRO are close to each other, and it was necessary to use 50 mM NaH_2_PO_4_ to improve the shape of the peaks. The applied conditions resulted in the satisfactory values of the dependent variables which are listed in Table 1. The most optimal conditions for separation of LEVO and MOXI separation are ACN: 1.5% TEA: 33.8 mM NaH_2_PO_4_ (27:36.5:36.5), the pH was 2.5 and the flow is 1 ml/min. In this case the content of ACN is higher than for the pair LEVO/CIPRO. The retention time for LEVO was 2.85 min. and for MOXI - 4.75 min. The application of MeOH would result in a long retention time of MOXI. The concentration of the phosphate is on the moderate level. The applied concentration of ion pair reagent did not exceed the value 2% which was found in the HPLC analysis of fluoroquinolones^13^. The chromatogram is presented in the Fig. 2. The values of the dependent variables for LEVO and MOXI separation are listed in Table 2 and they are similar to the calculated ones.

**Table 1:** The values of the dependent variables observed for LEVO and CIPRO at the optimal conditions.

Parameter	CIPRO		LEVO	
	Calculated values	Observed values	Calculated values	Observed values
Retention time	6.65	6.19	5.10	5.41
RRT^a^	1.10	1.14	0.85	0.87
Symmetry	1.18	1.24	1.16	1.27
Tailing factor	1.22	1.30	1.33	1.40
N^b^	5803	5608	4767	4985
Foley-Dorsey	5214	5402	4678	4612
R_S_	2.28	2.45	2.28	2.45
PW^c^	0.22	0.19	0.21	0.18

^a^RRT – relative retention time; ^b^N- the number of theoretical plates; ^c^PW – peak-width in half height.

**Table 2:** The values of the dependent variables observed for LEVO and MOXI at the optimal conditions.

Parameter	LEVO		MOXI	
	Calculated values	Observed values	Calculated values	Observed values
Retention time	3.22	2.85	5.40	4.75
RRT^a^	0.68	0.61	1.60	1.63
Symmetry	1.48	1.35	1.26	1.31
Tailing factor	1.32	1.44	1.15	1.33
N^b^	4871	4564	5318	5396
Foley-Dorsey	4712	4519	4697	4949
R_S_	7.54	7.86	7.54	7.86
PW^c^	0.11	0.10	0.17	0.15

^a^RRT – relative retention time; ^b^N- the number of theoretical plates; ^c^PW – peak-width in half height.

The peaks are fully resolved. The desired R_s_ value was higher than 2.0. The tailing and the symmetry factors are reduced and did not exceed the value 1.5. The content of the mobile phase provides a high capacity of the column - in both cases the number of theoretical plates exceeded the value 4000 (Tables 1 and 2). According to Guiliame *et al*. the capacity of the column depends mainly on the composition of the mobile phase^18^. The application of proper organic solvent, pH and ion pair reagents may improve the capacity of the column. The other factor that should be taken into an account is the size of the particles – the lower size is applied, the higher capacity is observed. The lower size of the particles generates also the higher pressure on the column. In our study, we used the column with the particle size 5 μm. Due to the optimized content of the mobile phase the efficiency of the column is high regardless the size of the grain.

The validation parameters such as precision and accuracy and obeyed the EMA guidelines. They did not exceed 15%. The linearity was tested for all analytes for the range 0.4–10.0 mg/l for CIPRO, 0.5–10.0 mg/ml for LEVO and 0.2–10.0 mg/l for MOXI. The lowest concentration for the calibration curve was the LLOQ (lower limit of quantitation) for each of analyte. The R^2^ value was above 0.999 and the Mandell’s fitting test excluded the quadratic model for the investigated concentrations ranges. The solutions remained stable in the temperature 2–8 °C and −20 °C, after storage for 24 h at the room temperature. (Supplementary Tables S5 and S6).The carry-over was not observed. The analytes were fully resolved.

**Figure 3:**
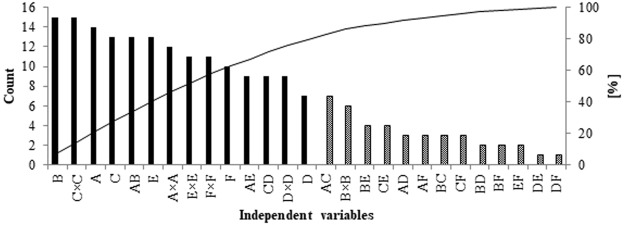
The Pareto Diagram for six-factor analysis (A- ACN, B- MeOH, C- TEA, D- NaH_2_PO_4_, E- pH, F- flow).

The Pareto Diagram for six-factor analysis (Fig. 3) indicates that in LEVO/CIPRO analysis the dominant factors are MeOH, ACN, TEA and pH and their quadratic terms. The most significant mutual interaction is ACN × MeOH which is necessary for separation of these substances. The other considerable interactions are ACN × pH and TEA × NaH_2_PO_4_. All the previously mentioned factors influence the dependent variables up to 80% (black color bars). The remain 20% are the interactions between different variables (dotted bars). The statistical analysis proved that in separation of fluoroquinolones, for which it is difficult to separate the peaks, the key role plays the proper pH, the content of organic phase and the proportion of ACN and MeOH, the content of the water-based phase and the concentration of ion-pair reagent.

**Figure 4:**
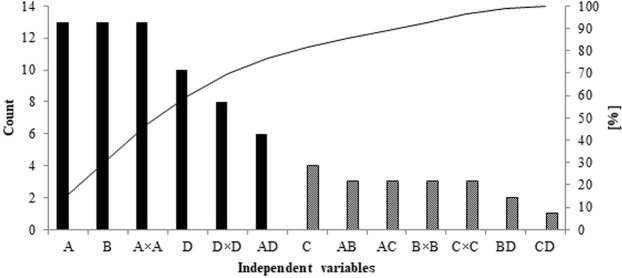
The Pareto Diagram for four-factor analysis (A-ACN, B-TEA, C-NaH_2_PO_4_, D-pH).

The Pareto Diagram for the four-factor analysis (Fig. 4) indicated ACN, TEA and ACN^2^ as predominant factors in LEVO/MOXI separation. pH, pH^2^ and the interaction ACN × pH are also significant. These variables influence the separation in almost 80%. The remained 20% are the phosphate concentration and the interactions between the factors.

## Conclusions

The BBD is a very useful statistic tool in the optimization of the separation of fluoroquinolones. It points out the statistically significant independent variables in single as well as their mutual interactions. It reveals the complexity of the chromatographic separation of fluoroquinolones and it is useful in the simultaneous optimization of different dependent variables essential for effective chromatographic analysis. The mutual interactions between the independent variables are described with the polynomial equations which makes it possible to predict the response under the applied conditions. The calculated values of the dependent variables with the polynomial equations and observed ones are similar. It confirmed the suitability of the applied design.

## Material and Methods

### Reagents

Acetonitrile (ACN), methanol (MeOH), triethylamine (TEA), dichloromethane (DCM) were purchased by Merck (Germany). Levofloxacin (LEVO), Ciprofloxacin (CIPRO), orthophosphoric acid were purchased by Fluka (Germany), Moxifloxacin (MOXI) was purchased by SantaCruz Biotechnology (USA). Sodium monophosphate was purchased by Sigma-Aldrich (Germany). All reagents were of HPLC grade.

### The chromatographic procedure

The 20 µL of the mixture of LEVO/MOXI or LEVO/CIPRO was injected into the chromatographic system. The concentration of each analyte was 10 mg/l. The analytes were detected with UV detector (detection wavelength λ = 280 nm), the temperature was ambient. The elution was isocratic. The RP –- 18 LiChroCART column (250 × 4 mm, 5 μm, Merck Germany) with Purospher RP – 18 guard column (4 × 4 mm, 5 μm, Merck Germany) were applied for chromatographic separation. The data were analysed with OpenLab ChemStation ver.A.01.05 Software.

### The optimization of the chromatographic procedure

The four- and six-factor designs were considered for LEVO/MOXI and LEVO/CIPRO analyses respectively. The following independent variables were considered in LEVO/MOXI analysis: % ACN, % TEA, the concentration of NaH_2_PO_4_, pH. In an analysis of LEVO/CIPRO, there were also considered two extra parameters: %MeOH and flow of the mobile phase. The intervals between the levels are equal, and they are presented in Tables 3 and 4 for four and six independent variables, respectively.

**Table 3:** The levels of the independent variables in the analysis of LEVO and MOXI.

Factor		Level		
		−1	0	1
A	ACN [%]	20	30	40
B	TEA [%]	0.5	1.0	1.5
C	NaH_2_PO_4_ [mM]	10	30	50
D	pH	2	3	4

**Table 4:** The levels of the independent variables for CIPRO and LEVO analysis.

Factor		Level		
		−1	0	1
A	ACN [%]	1	7	13
B	MeOH [%]	17	23	29
C	TEA [%]	0.5	1.0	1.5
D	NaH_2_PO_4_ [mM]	10	30	50
E	pH	2	3	4
F	flow [ml/min]	0.7	1.0	1.3

Using the experimental design shown in the Supplementary Tables S7 and S8 a set of experiments was performed. The retention time, the relative retention time, the symmetry, tailing factor, the number of theoretical plates, Foley-Dorsey parameter, the peak width in half height, the resolution between peaks were taken into consideration as dependent variables.

### Statistical analysis

The statistical analysis was done by Design Expert ver. 11 and Statistica ver. 13.1. The ANOVA test was applied. The statistically not significant parameters and their interactions were omitted. They are presented in the Supplementary Tables S3a–h and S4a–h. The *p < 0.05* was considered as statistically significant.

The R^2^ close to 1 implies a better fit design to experimental data which is also supported with the R^2^ adjusted. The adjusted R^2^ value also explains the suitability of the design. It increases with the addition of independent variables which are significant to a dependent variable. If the non-significant variables are added into the design the adjusted R^2^ value decrease contrary to the R^2^ value that increases with the introduction of the new variables regardless of their importance. The gap between R^2^ and adjusted R^2^ should be as small as possible and not exceed 0.2^19^. The *p-values* for four- and six-factor design were < 0.0001 which implies that there is only a 0.01% chance that *F-values* is significant due to the noise. The importance of *F-value* implies the significance of the variable in single or in interaction. The higher *F-value* is observed, the more significant factor is.

The distribution of the data was normal (*p < 0.05*) and it was confirmed with the Shapiro-Wilk test.

### Appendix

Supplementary Information: Part A- The tables with the parameters of the statistical analysis; Part B - The polynomial equations for the parameters of the chromatographic separation; Part C -The RSM diagrams for the six- and four factor BBD.

## Supplementary information

Supplementary information

## References

[1] Hibbert DB (2012). Experimental design in chromatography: a tutorial review. J. Chromatogr. B Analyt. Technol. Biomed. Life Sci..

[2] de Almeida Borges VR, Ribeiro AF, de Souza Anselmo C, Cabral LM, de Sousa VP (2013). Development of a high performance liquid chromatography method for quantification of isomers β-caryophyllene and α-humulene in copaiba oleoresin using the Box-Behnken design. J. Chromatogr. B Analyt. Technol. Biomed. Life Sci..

[3] Kumar V, Bhalla A, Rathore AS (2014). Design of experiments applications in bioprocessing: concepts and approach. Biotechnol. Prog..

[4] Czyrski A, Kondys K, Szałek E, Karbownik A, Grześkowiak E (2015). The pharmacokinetic interaction between levofloxacin and sunitinib. Pharmacol Rep.

[5] Wiczling P, Kaliszan R (2016). How Much Can We Learn from a Single Chromatographic Experiment? A Bayesian Perspective. Anal. Chem..

[6] Szerkus O (2016). Ultra-high performance liquid chromatographic determination of levofloxacin in human plasma and prostate tissue with use of experimental design optimization procedures. J. Chromatogr. B Analyt. Technol. Biomed. Life Sci..

[7] Box GEP, Behnken D (1960). Some New Three Level Designs for the Study of Quantitative Variables. Technometrics.

[8] Ferreira SLC (2007). Statistical designs and response surface techniques for the optimization of chromatographic systems. J Chromatogr A.

[9] Mirza T, Tan HS (2001). Determination of captopril in pharmaceutical tablets by anion-exchange HPLC using indirect photometric detection; a study in systematic method development. J Pharm Biomed Anal.

[10] Kalariya PD, Namdev D, Srinivas R, Gananadhamu S (2017). Application of experimental design and response surface technique for selecting the optimum RP-HPLC conditions for the determination of moxifloxacin HCl and ketorolac tromethamine in eye drops. Journal of Saudi Chemical Society.

[11] Ferreira SLC (2007). Box-Behnken design: an alternative for the optimization of analytical methods. Anal. Chim. Acta.

[12] Chamseddin C, Jira TH (2011). Comparison of the chromatographic behavior of levofloxacin, ciprofloxacin and moxifloxacin on various HPLC phases. Pharmazie.

[13] Czyrski A (2017). Analytical Methods for Determining Third and Fourth Generation Fluoroquinolones: A Review. Chromatographia.

[14] Zhang J, Wang Q, Kleintop B, Raglione T (2014). Suppression of peak tailing of phosphate prodrugs in reversed-phase liquid chromatography. J Pharm Biomed Anal.

[15] Vybíralová Z, Nobilis M, Zoulova J, Květina J, Petr P (2005). High-performance liquid chromatographic determination of ciprofloxacin in plasma samples. Journal of Pharmaceutical and Biomedical Analysis.

[16] Petruczynik A (2012). Effect of Ionic Liquid Additives to Mobile Phase on Separation and System Efficiency for HPLC of Selected Alkaloids on Different Stationary Phases. J Chromatogr Sci.

[17] Kasagić I (2013). Chemometrically assissted optimization and validation of RP-HPLC method for the analysis of itraconazole and its impurities. Acta Pharm.

[18] Guillaume Y, Guinchard C (1994). Study and Optimization of Column Efficiency in HPLC: Comparison of Two Methods for Separating Ten Benzodiazepines. Journal of Liquid Chromatography.

[19] Nam S-N, Cho H, Han J, Her N, Yoon J (2018). Photocatalytic degradation of acesulfame K: Optimization using the Box–Behnken design (BBD). Process Safety and Environmental Protection.

