# Supplementary material for: The application of Box-Behnken-Design in the optimization of HPLC separation of fluoroquinolones
Source: Sci Rep. 2019 Dec 19;9:19458. doi: 10.1038/s41598-019-55761-z (PMC6923357; doi:10.1038/s41598-019-55761-z)
Supplement: Supplementary file 1 — Supplementary information [file 41598_2019_55761_MOESM1_ESM.pdf]

The application of Box-Behnken-Design in the optimization of HPLC separation of fluoroquinolones

Andrzej Czyrski, Justyna Sznura

SUPPLEMENTARY INFORMATION

PART A – The tables with the parameters of the statistical analysis

- **The analysis of the model**

Table S.1. The six-factor model analysis for LEVO and CIPRO.

Response	*p-value*	R^2	R^2 adjusted
tr[a] cipro	<0.0001	0.9877	0.9739
tr levo	<0.0001	0.9891	0.9758
RRT[b] cipro	<0.0001	0.9864	0.9705
RRT levo	<0.0001	0.9855	0.9677
Symmetry cipro	<0.0001	0.9866	0.9666
Symmetry levo	<0.0001	0.9911	0.9768
Tf[c] cipro	<0.0001	0.9864	0.9672
Tf levo	<0.0001	0.9778	0.9628
N[d] cipro	<0.0001	0.9628	0.9429
N levo	<0.0001	0.9506	0.9437
FD[e] cipro	<0.0001	0.9722	0.9579
FD levo	<0.0001	0.9597	0.9616
Rs	<0.0001	0.9727	0.9644
PW[f] cipro	<0.0001	0.9622	0.9482
PW levo	<0.0001	0.9725	0.9607

[a]t_r- retention time; [b]RRT- Relative retention time; [c]Tf – tailing factor; [d]N – the number of theoretical plates [e]FD-Foley-Dorsey; [f]PW – peak width in half height

Table S.2. The four-factor model analysis for LEVO and MOXI.

Response	*p-value*	R^2	R^2 adjusted
tr[a] moxi	<0.0001	0.9992	0.9990
tr levo	<0.0001	0.9993	0.9978
RRT[b] moxi	<0.0001	0.9980	0.9974
RRT levo	<0.0001	0.9926	0.9906
Symmetry moxi	<0.0001	0.9872	0.9808
Symmetry levo	<0.0001	0.9912	0.9799
Tf[c] moxi	<0.0001	0.9599	0.9476
Tf levo	<0.0001	0.9952	0.9910
N[d] moxi	<0.0001	0.9841	0.9618
N levo	<0.0001	0.9964	0.9919
FD[e] moxi	<0.0001	0.9888	0.9430
FD levo	<0.0001	0.9762	0.9414
Rs	<0.0001	0.9897	0.9877
PW[f] moxi	<0.0001	0.9974	0.9958
PW levo	<0.0001	0.9766	0.9626

[a]t_r- retention time; [b]RRT- Relative retention time; [c]Tf – tailing factor; [d]N – the number of theoretical plates [e]FD-Foley-Dorsey; [f]PW – peak width in half height

- **The ANOVA test parameters for six-factor analysis**

Table S.3a. The ANOVA test for retention time of CIPRO and LEVO.

Parameter	CIPRO		LEVO	
	F	*p*	*F*	*P*
Model	169.00	<0.0001	131.18	<0.0001
A-ACN	1292.74	<0.0001	898.65	<0.0001
B-MeOH	454.78	<0.0001	370.45	<0.0001
C-TEA	5.48	0.0246	15.24	0.0004
E-pH	0.69	0.4099	9.67	0.0036
F-flow	325.17	<0.0001	300.38	<0.0001
AB	72.30	<0.0001	55.63	<0.0001
AF	63.59	<0.0001	31.49	<0.0001
BF	15.55	0.0003	15.81	0.0003
A^2	156.64	<0.0001	95.72	<0.0001
B^2	5.21	0.0282	5.24	0.0281
C^2	21.56	<0.0001	22.84	<0.0001
E^2	4.77	0.0352	5.66	0.0228
F^2	11.64	0.0015	9.78	0.0035

Table S.3b. The ANOVA test for relative retention time of CIPRO and LEVO.

Parameter	CIPRO		LEVO	
	F	*p*	*F*	*p*
Model	134.43	<0.0001	152.54	<0.0001
A-ACN	1037.87	<0.0001	1389.53	<0.0001
B-MeOH	209.61	<0.0001	264.93	<0.0001
C-TEA	64.44	<0.0001	48.57	<0.0001
D-NaH_2PO_4	16.52	0.0005	11.83	0.0015
E-pH	154.86	<0.0001	154.76	<0.0001
F-flow	0.31	0.5783	1.38	0.2476
AB	25.81	<0.0001	12.21	0.0013
AE	9.10	0.0046	10.68	0.0024
A^2	100.45	<0.0001	67.01	<0.0001
C^2	17.41	0.0002	10.41	0.0027
D^2	5.16	0.0290	6.78	0.0133
E^2	11.42	0.0017	13.77	0.0007
F^2	8.46	0.0061	8.30	0.0067

Table S.3c. The ANOVA test for symmetry of CIPRO and LEVO peaks.

Parameter	CIPRO		Parameter	LEVO	
	F	*p*		*F*	*p*
Model	70.15	<0.0001	Model	89.72	<0.0001
A-ACN	0.41	0.5265	A-ACN	661.65	<0.0001
B-MeOH	13.78	0.0009	B-MeOH	82.75	<0.0001
C-TEA	95.10	<0.0001	C-TEA	27.54	<0.0001
D-NaH_2PO_4	18.33	0.0002	D-NaH_2PO_4	7.04	0.0142
E-pH	200.39	<0.0001	E-pH	72.28	<0.0001
F- flow	2.88	0.1010	F- flow	22.91	<0.0001
AB	13.32	0.0011	AB	21.60	0.0001
BC	169.78	<0.0001	AC	116.96	<0.0001
BD	7.52	0.0105	AD	28.67	<0.0001
BE	5.54	0.0259	AE	73.39	<0.0001
CD	31.92	<0.0001	BC	146.61	<0.0001
DF	4.78	0.0373	BE	23.44	<0.0001
A^2	47.82	<0.0001	CD	36.52	<0.0001
C^2	197.98	<0.0001	CE	7.46	0.0119
D^2	9.83	0.0040	CF	6.26	0.0199
E^2	23.84	<0.0001	EF	16.52	0.0005
F^2	369.50	<0.0001	B^2	118.01	<0.0001
			C^2	195.51	<0.0001
			D^2	68.90	<0.0001
			E^2	116.23	<0.0001
			F^2	50.20	<0.0001

Table S.3d. The ANOVA test for the tailing factor of CIPRO and LEVO peaks.

Parameter	CIPRO		Parameter	LEVO	
	F	*p*		*F*	*p*
Model	64.40	<0.0001	Model	64.96	<0.0001
A-ACN	21.67	<0.0001	A-ACN	641.07	<0.0001
B-MeOH	18.75	0.0002	B-MeOH	76.52	<0.0001
C-TEA	82.23	<0.0001	C-TEA	24.68	<0.0001
D-NaH_2PO_4	8.60	0.0066	D-NaH_2PO_4	17.82	0.0002
E-pH	90.25	<0.0001	E-pH	12.70	0.0013
F- flow	2.10	0.1584	F- flow	10.11	0.0036
AB	19.55	0.0001	AB	8.01	0.0085
AD	6.49	0.0166	AC	60.93	<0.0001
AE	4.39	0.0453	AD	22.69	<0.0001
BD	7.41	0.0110	AE	35.76	<0.0001
BE	4.78	0.0374	BC	94.00	<0.0001
CD	18.84	0.0002	CD	24.82	<0.0001
CE	209.87	<0.0001	DE	10.28	0.0034
A^2	13.39	0.0010	EF	10.84	0.0027
B^2	27.64	<0.0001	B^2	30.34	<0.0001
C^2	215.39	<0.0001	C^2	95.55	<0.0001
E^2	36.58	<0.0001	D^2	57.84	<0.0001
F^2	219.20	<0.0001	E^2	70.66	<0.0001
			F^2	30.58	<0.0001

Table S.3e. The ANOVA test for peak width in half height of CIPRO and LEVO peaks.

Parameter	CIPRO		Parameter	LEVO	
	F	*p*		*F*	*p*
Model	68.52	<0.0001	Model	82.40	<0.0001
A-ACN	499.26	<0.0001	A-ACN	690.50	<0.0001
B-MeOH	150.25	<0.0001	B-MeOH	175.03	<0.0001
C-TEA	43.80	<0.0001	C-TEA	66.05	<0.0001
D-NaH_2PO_4	1.38	0.2482	D-NaH_2PO_4	2.72	0.1081
E-pH	17.74	0.0002	E-pH	70.47	<0.0001
F- flow	86.62	<0.0001	F- flow	151.31	<0.0001
AB	26.54	<0.0001	AB	35.88	<0.0001
AC	15.48	0.0004	AC	31.09	<0.0001
CD	11.01	0.0021	AE	22.32	<0.0001
CE	8.58	0.0060	AF	35.40	<0.0001
A^2	61.89	0.0374	CD	17.57	0.0002
C^2	54.36	<0.0001	CE	9.61	0.0038
D^2	11.70	0.0016	CF	5.94	0.0200
			A^2	67.46	<0.0001
			C^2	46.91	<0.0001

Table S.3f. The number of theoretical plates for CIPRO and LEVO.

Parameter	CIPRO		Parameter	LEVO	
	F	*p*		*F*	*p*
Model	31.22	<0.0001	Model	21.52	<0.0001
A-ACN	7.41	0.0112	A-ACN	66.95	<0.0001
B-MeOH	68.27	<0.0001	B-MeOH	6.96	0.0116
C-TEA	0.00	0.9953	C-TEA	31.08	<0.0001
D-NaH_2PO_4	0.22	0.6417	D-NaH_2PO_4	0.71	0.4035
E-pH	8.56	0.0069	E-pH	63.45	<0.0001
F- flow	38.78	<0.0001	F- flow	4.83	0.0336
AC	19.99	0.0001	AC	80.18	0.0066
CD	74.69	<0.0001	CD	17.03	0.0002
A^2	49.38	<0.0001	C^2	5.18	0.0280
B^2	43.75	<0.0001	D^2	12.23	0.0011
C^2	9.50	0.0047	F^2	5.09	0.0293
D^2	30.43	<0.0001			

Table S.3g. The Foley-Dorsey parameter for CIPRO and LEVO.

Parameter	CIPRO		Parameter	LEVO	
	F	*p*		*F*	*p*
Model	38.85	<0.0001	Model	33.40	<0.0001
A-ACN	20.40	<0.0001	A-ACN	304.90	<0.0001
B-MeOH	130.59	<0.0001	B-MeOH	15.23	0.0005
C-TEA	20.80	<0.0001	C-TEA	2.06	0.1611
D-NaH_2PO_4	2.69	0.1114	E-pH	2.04	0.1631
E-pH	63.82	<0.0001	F- flow	4.26	0.0478
F- flow	49.66	<0.0001	AB	38.47	<0.0001
AB	77.82	<0.0001	AE	8.71	0.0061
AE	26.28	<0.0001	BE	4.65	0.0391
A^2	39.59	<0.0001	CE	4.54	0.0415
C^2	6.81	0.0138	CF	4.93	0.0340
D^2	39.19	<0.0001	A^2	65.15	<0.0001
E^2	20.15	<0.0001	C^2	7.16	0.0120
F^2	57.22	<0.0001	E^2	8.19	0.0076
			F^2	6.41	0.0168

Table S.3h.The R_s value for CIPRO and LEVO.

Parameter	*F*	*p*
Model	112.62	<0.0001
A-ACN	623.62	<0.0001
B-MeOH	250.55	<0.0001
C-TEA	73.26	<0.0001
D-NaH_2PO_4	6.32	0.0163
E-pH	183.86	<0.0001
AB	19.71	<0.0001
AC	14.17	0.0006
AE	31.22	<0.0001
CD	15.26	0.0004
A^2	36.02	<0.0001
C^2	18.63	0.0001
E^2	6.58	0.0144

- **The ANOVA test parameters for four-factor analysis**

Table S.4a. The ANOVA test retention time for LEVO and MOXI.

Parameter	LEVO		Parameter	MOXI	
	F	*p*		*F*	*p*
Model	493.81	<0.0001	Model	2621.58	<0.0001
A-ACN	1397.05	<0.0001	A-ACN	6505.83	<0.0001
C-NaH_2PO_4	5.74	0.0265	B-TEA	5.35	0.0315
AC	16.77	0.0006	A^2	2515.45	<0.0001
A^2	553.3	<0.0001			

Table S.4b. The ANOVA test relative retention time for LEVO and MOXI.

Parameter	LEVO		MOXI	
	F	*p*	*F*	*p*
Model	485.40	<0.0001	1761.42	<0.0001
A-ACN	2115.77	<0.0001	6657.95	<0.0001
B-TEA	29.17	<0.0001	50.54	<0.0001
D-pH	0.29	0.5990	0.21	0.6506
A^2	124.57	<0.0001	1559.22	<0.0001
D^2	6.04	0.0244	16.63	0.0007

Table S.4c. The ANOVA test for symmetry of LEVO and MOXI peaks.

Parameter	LEVO		Parameter	MOXI	
	F	*p*		*F*	*p*
Model	87.73	<0.0001	Model	153.99	<0.0001
A-ACN	474.16	<0.0001	A-ACN	102.14	<0.0001
B-TEA	6.85	0.0345	B-TEA	263.62	<0.0001
C-NaH_2PO_4	18.75	0.0034	C-NaH_2PO_4	5.53	0.0406
D- pH	0.19	0.6738	D- pH	366.82	<0.0001
AD	31.78	0.0008	BD	23.22	0.0007
CD	32.06	0.0088			
A^2	32.06	0.0008			
B^2	11.03	0.0128			
D^2	147.78	<0.0001			

Table S.4d. The ANOVA test for tailing of LEVO and MOXI peaks.

Parameter	LEVO		Parameter	MOXI	
	F	*p*		*F*	*p*
Model	235.10	<0.0001	Model	77.80	<0.0001
A- ACN	469.02	<0.0001	A- ACN	237.73	<0.0001
B- TEA	215.24	<0.0001	B- TEA	30.66	<0.0001
C- NaH_2PO_4	7.37	0.0238	D- pH	52.75	<0.0001
D- pH	126.61	<0.0001	A^2	115.75	<0.0001
AB	78.94	<0.0001			
A^2	292.23	<0.0001			
C^2	186.68	<0.0001			
D^2	131.68	<0.0001			

Table S.4e. The ANOVA test of peak width in half-height of LEVO and MOXI.

Parameter	LEVO		Parameter	MOXI	
	F	*p*		*F*	*p*
Model	69.58	<0.0001	Model	634.66	<0.0001
A-ACN	33.43	<0.0001	A-ACN	3621.24	<0.0001
B- TEA	1.46	0.2456	B-TEA	32.17	<0.0001
C- NaH_2PO_4	1.07	0.3171	C- NaH_2PO_4	1.91	0.1872
D-pH	33.03	<0.0001	D-pH	34.99	<0.0001
AB	16.95	0.0009	AC	6.00	0.0270
AC	3.60	0.0773	AD	25.52	0.0001
AD	8.63	0.0102	A^2	1882.05	<0.0001
BD	3.33	0.0880	B^2	6.16	0.0254
A^2	524.72	<0.0001	D^2	13.60	0.0022

Table S.4f. The ANOVA test for a number of theoretical plates for LEVO and MOXI.

Parameter	LEVO		Parameter	MOXI	
	F	*p*		*F*	*p*
Model	221.58	<0.0001	Model	106.56	<0.0001
A- ACN	604.84	<0.0001	A-ACN	330.97	<0.0001
B- TEA	29.55	0.0006	B-TEA	65.20	<0.0001
C- NaH_2PO_4	0.44	0.5252	D-pH	91.17	<0.0001
D- pH	266.06	<0.0001	AD	13.30	0.0018
AB	48.72	0.0001	A^2	132.15	<0.0001
BD	33.78	0.0004	D^2	23.13	0.0001
A^2	1013.29	<0.0001			
B^2	20.59	0.0019			
C^2	66.69	<0.0001			
D^2	32.38	0.0005			

Table S.4g. The ANOVA test for Foley-Dorsey for LEVO and MOXI.

Parameter	LEVO		Parameter	MOXI	
	F	*p*		*F*	*p*
Model	23.77	<0.0001	Model	19.41	<0.0001
A- ACN	2.92	0.1031	A- ACN	0.08	0.7864
B-TEA	7.74	0.0115	B-TEA	33.02	<0.0001
D-pH	19.27	0.0003	C- NaH_2PO_4	0.18	0.6731
A^2	65.13	<0.0001	D-pH	71.35	<0.0001
			AD	11.58	0.0034
			C^2	3.60	0.0751
			D^2	12.57	0.0025

Table S.4h. The ANOVA test for R_s value for LEVO and MOXI.

Parameter	*F*	*p*
Model	481.78	<0.0001
A-ACN	1744.13	<0.0001
D-pH	13.87	0.0013
AD	10.44	0.0042
A^2	158.69	<0.0001

- **The validation parameters**

Table S.5. The precision and accuracy results for CIPRO, LEVO and MOXI

Nominal concentration [mg/ml]	Inter-day assay		Intra-day assay	
	Precision [%]	Accuracy [%]	Precision [%]	Accuracy [%]
CIPROFLOXACIN				
0.4	9.62	5.70	3.92	5.41
2	3.65	5.96	4.21	0.71
4	4.94	7.02	1.44	0.18
8	2.73	0.81	1.42	5.32
LEVOFLOXACIN				
0.5	8.78	5.05	7.33	8.44
2	7.69	6.14	4.38	4.87
4	3.31	1.68	2.84	2.70
8	3.62	0.27	1.39	3.84
MOXIFLOXACIN				
0.2	8.27	6.67	5.74	5.21
1	8.89	8.95	6.31	5.20
4	2.99	3.19	3.62	3.42
8	3.88	1.16	7.49	1.62

Table S.6. The stability of the analytes

Experimental conditions	Concentration [mg/l]	
	2	8
**CIPROFLOXACIN**		
After storage at room temperature for 24 h		
Mean assayed value [mg/l]	2.12	7.89
Accuracy [%RE]	6.50	4.22
After three freeze-thaw cycles		
Mean assayed value [mg/l]	2.11	8.06
Accuracy [%RE]	2.76	0.50
After storage for three months at the temperature -20°C		
Mean assayed value [mg/l]	2.09	8.06
Accuracy [%RE]	5.08	5.32
**LEVOFLOXACIN**		
After storage at room temperature for 24 h		
Mean assayed value [mg/l]	2.13	8.31
Accuracy [%RE]	1.42	2.00
After three freeze-thaw cycles		
Mean assayed value [mg/l]	1.94	7.84
Accuracy [%RE]	2.51	2.03
After storage for three months at the temperature -20°C		
Mean assayed value [mg/l]	2.05	8.19
Accuracy [%RE]	2.50	2.63
**MOXIFLOXACIN**		
After storage at room temperature for 24 h		
Mean assayed value [mg/l]	2.05	8.10
Accuracy [%RE]	2.50	1.25
After three freeze-thaw cycles		
Mean assayed value [mg/l]	2.07	8.04
Accuracy [%RE]	3.50	1.36
After storage for three months at the temperature -20°C		
Mean assayed value [mg/l]	2.12	8.21
Accuracy [%RE]	6.00	2.62

- **The designs of the experiments**

Table S.7. The four-factor Box-Behnken Design for LEVO and MOXI analysis.

FACTOR			
A	B	C	D
±1	±1	0	0
0	0	±1	±1
0	0	0	0
±1	0	0	±1
0	±1	±1	0
0	0	0	0
±1	0	±1	0
0	±1	0	±1
0	0	0	0

Table S.8. The six-factor Box-Behnken Design for CIPRO and LEVO analysis

FACTOR					
A	B	C	D	E	F
±1	±1	0	±1	0	0
0	±1	±1	0	±1	0
0	0	±1	±1	0	±1
±1	0	0	±1	±1	0
0	±1	0	0	±1	±1
±1	0	±1	0	0	±1
0	0	0	0	0	0

PART B – The polynomial equations for the parameters of the chromatographic separation

- **The equations for six-factor analysis.**

$t_{R\ CIPRO}$ = 6.54 – 9.99 ACN – 4.19 MeOH – 0.492 TEA + 0.175 pH – 3.79 flow + 8.81 ACN × MeOH + 2.90 ACN × flow + 1.44 MeOH × flow + 4.00 ACN^2 + 0.813 $MeOH^2$ + 1.49 TEA^2 + 0.764 pH^2 + 1.09 $flow^2$

$t_{R\ LEVO}$ = 5.97 – 6.15 ACN – 0.34 MeOH – 0.718 × TEA + 0.573 pH – 3.29 flow + 3.03 ACN×MeOH + 1.89 ACN×flow + 1.35 MeOH×flow + 2.71 ACN^2 + 0.703 $MeOH^2$ + 1.35 TEA^2 + 0.707 pH^2 + 0.893 $flow^2$

RRT_{CIPRO}= 1.17 – 0.097 ACN – 0.043 MeOH + 0.023 TEA + 0.012 NaH_2PO_4 – 0.037 pH + 0.0016 flow + 0.027 ACN×MeOH + 0.0161 ACN×pH + 0.0434 ACN^2 – 0.0184 TEA^2 – 0.0098 $NaH_2PO_4{}^2$ – 0.0144 pH^2 + 0.0127 $flow^2$

RRT_{LEVO} = 0.85 + 0.069 ACN + 0.031 MeOH – 0.0138 TEA – 0.0065 NaH_2PO_4 + 0.0238 pH – 0.0022 flow – 0.0118 ACN×MeOH – 0.0101 ACN×pH – 0.0229 ACN^2 + 0.0095 TEA^2 + 0.0073 $NaH_2PO_4{}^2$ + 0.0101 pH^2 – 0.0083 $flow^2$

Sym_{CIPRO} = 1.19 + 0.0051 ACN + 0.0325 MeOH – 0.093 TEA – 0.039 NaH_2PO_4 + 0.130 pH – 0.013 flow + 0.052 ACN×MeOH + 0.24 MeOH×TEA + 0.039 MeOH×NaH_2PO_4 + 0.027 MeOH×pH + 0.098 TEA×NaH_2PO_4 – 0.033 NaH_2PO_4×flow – 0.088 ACN^2 + 0.188 TEA^2 – 0.041 $NaH_2PO_4{}^2$ + 0.058 pH^2 – 0.25 $flow^2$

Sym_{LEVO} = 1.55 – 0.207 ACN – 0.102 MeOH – 0.057 TEA – 0.027 NaH_2PO_4 + 0.098 pH – 0.044 flow – 0.066 ACN×MeOH + 0.123 ACN×TEA + 0.060 ACN×NaH_2PO_4 – 0.177 ACN×pH – 0.257 MeOH×TEA – 0.062 MeOH×pH – 0.122 TEA×NaH_2PO_4 + 0.056 TEA×pH + 0.028 TEA×flow – 0.065 pH×flow – 0.129 $MeOH^2$ – 0.189 TEA^2 – 0.097 $NaH_2PO_4{}^2$ – 0.150 pH^2 – 0.103 $flow^2$

$T_{f\ CIPRO}$= 1.16 + 0.0070 ACN + 0.067 MeOH – 0.157 TEA – 0.0504 NaH_2PO_4 + 0.154 pH – 0.0209 flow + 0.119 ACN×MeOH + 0.049 ACN×NaH_2PO_4 + 0.055 ACN×pH + 0.073 MeOH×NaH_2PO_4 + 0.0406 MeOH × pH + 0.130 TEA×NaH_2PO_4 – 0.476 TEA×pH – 0.080 ACN^2 + 0.121 $MeOH^2$ + 0.356 TEA^2 + 0.136 pH^2 – 0.346 $flow^2$

$T_{f\ LEVO}$ = 1.38 – 0.258 ACN – 0.101 MeOH – 0.062 TEA – 0.048 NaH_2PO_4 + 0.044 pH – 0.035 flow – 0.050 ACN × MeOH + 0.138 ACN×TEA + 0.060 ACN×NaH_2PO_4 – 0.106 ACN×pH – 0.237 MeOH×TEA – 0.113 TEA×NaH_2PO_4 – 0.057 NaH_2PO_4×pH – 0.064 pH×flow – 0.088 $MeOH^2$ – 0.180 TEA^2 – 0.125 $NaH_2PO_4{}^2$ – 0.135 pH^2 – 0.099 $flow^2$

N_{CIPRO} = 4132.33 – 121.72 ACN – 364.30 MeOH – 0.268 TEA + 22.06 NaH_2PO_4 – 140.19 pH – 269.84 flow – 320.59 ACN×TEA – 681.09 TEA×NaH_2PO_4 + 455.65 ACN^2 + 445.4 $MeOH^2$ – 202.03 TEA^2 + 359.22 $NaH_2PO_4{}^2$

N_{LEVO} = 4243.87 + 378.50 ACN + 122.05 MeOH + 257.88 TEA + 39.04 NaH_2PO_4 – 368.49 pH – 101.65 flow – 229.13 ACN×TEA – 330.63 TEA×NaH_2PO_4 – 155.53 TEA^2 + 223.51 $NaH_2PO_4{}^2$ – 154.19 $flow^2$

FD_{CIPRO} = 1854.32 – 196.95 ACN – 442.25 MeOH +168.66 TEA + 63.03 NaH_2PO_4 – 347.16 pH – 284.60 flow – 577.07 ACN×MeOH + 400.26 ACN×pH + 370.04 ACN^2 – 152.47 TEA^2 + 377.96 $NaH_2PO_4^2$ – 253.66 pH^2 – 463.82 $flow^2$

FD_{LEVO} = 4595.36 + 872.29 ACN + 159.26 MeOH – 66.24 TEA + 67.46 pH – 88.87 flow – 414.50 ACN×MeOH + 272.92 ACN × pH + 117.08 MeOH×pH – 182.22 TEA×pH – 124.74 TEA × flow + 548.77 ACN^2 + 179.30 TEA^2 + 179.90 pH^2 – 165.56 $flow^2$

R_S = 1.79 – 0.79 ACN – 0.55 MeOH + 0.31 TEA + 0.099 NaH_2PO_4 – 0.475 pH + 0.26 ACN × MeOH – 0.24 ACN × TEA + 0.33 ACN × pH – 0.25 TEA × NaH_2PO_4 + 0.30 ACN^2 – 0.218 TEA^2 – 0.128 pH^2

Peak width $_{CIPRO}$ = 0.25 – 0.360 ACN – 0.190 MeOH – 0.097 TEA – 0.017 NaH_2PO_4 + 0.060 pH – 0.142 flow + 0.154 ACN×MeOH + 0.114 ACN×TEA + 0.0798 TEA×NaH_2PO_4 – 0.070 TEA × pH + 0.18 ACN^2 + 0.15 TEA^2 – 0.075 $NaH_2PO_4^2$

Peak width $_{LEVO}$ = 0.38 – 0.480 ACN – 0.250 MeOH – 0.140 TEA – 0.029 NaH_2PO_4 + 0.140 pH – 0.213 flow + 0.216 ACN×MeOH + 0.163 ACN×TEA – 0.138 ACN×pH + 0.174 ACN×flow + 0.123 TEA×NaH_2PO_4 – 0.091 TEA×pH + 0.050 TEA×flow + 0.199 ACN^2 + 0.163 TEA^2

- **The equations for four-factor analysis.**

$t_{R\ MOXI}$ = 3.44 – 5.69 ACN – 0.16 TEA + 4.64 ACN^2

$t_{R\ LEVO}$ = 2.71 – 1.29 ACN + 0.083 NaH_2PO_4 – 0.24 ACN × NaH_2PO_4 + 1.092 ACN^2

RRT_{MOXI} = 1.35 – 0.887 ACN – 0.077 TEA + 0.0047 pH + 0.568 ACN^2 + 0.0579 pH^2

RRT_{LEVO} = 0.74 + 0.285 ACN + 0.033 TEA – 0.0031 pH – 0.0914 ACN^2 – 0.0199 pH^2

Sym_{MOXI} = 1.48 + 0.105 ACN – 0.1405 TEA – 0.020 NaH_2PO_4 + 0.144 pH – 0.057 TEA×pH

Sym_{LEVO} = 1.47 – 0.19 ACN + 0.032 TEA + 0.032 NaH_2PO_4 + 0.0044 pH – 0.104 ACN×pH – 0.149 NaH_2PO_4×pH – 0.069 ACN^2 – 0.0404 TEA^2 + 0.14 pH^2

$T_{f\ MOXI}$ = 1.64 + 0.476 ACN – 0.144 TEA + 0.181 pH + 0.403 ACN^2

$T_{f\ LEVO}$ = 1.55 – 0.345 ACN – 0.215 TEA + 0.0389 NaH_2PO_4 + 0.1513 pH + 0.2745 ACN×TEA – 0.326 ACN^2 + 0.255 $NaH_2PO_4^2$ + 0.212 pH^2

N_{MOXI} = 4401.67 – 1029.83 ACN + 457.08 TEA – 540.50 pH + 357.62 ACN×pH – 891.06 ACN^2 – 372.81 pH^2

N_{LEVO} = 4457.67 – 1186.53 ACN + 325.88 TEA + 34.35 NaH_2PO_4 – 874.41 pH – 641.46 ACN×TEA + 539.89 TEA×pH – 2029.78 ACN^2 – 339.68 TEA^2 – 569.84 $NaH_2PO_4^2$ – 365.33 pH^2

FD_{MOXI} = 3910.17 – 26.67 ACN + 556.67 TEA + 41.58 NaH_2PO_4 – 818.25 pH – 571.00 ACN × pH + 251.50 $NaH_2PO_4^2$ – 470.25 pH^2

$$\mathbf{FD}_{\mathbf{LEVO}} = 4063.50 + 317.54\ ACN + 517.33\ TEA - 816.00\ pH - 2012.79\ ACN^2$$

$$\mathbf{R_s} = 4.77 - 6.90\ ACN - 0.616\ pH + 0.925\ ACN \times pH + 2.79\ ACN^2$$

$$\mathbf{PW_{moxi}} = 0.11 - 0.200\ ACN - 0.0189\ TEA + 0.0046\ NaH_2PO_4 + 0.020\ pH - 0.014\ ACN{\times}NaH_2PO_4 - 0.029\ ACN{\times}pH + 0.204\ ACN^2 + 0.0117\ TEA^2 + 0.017\ pH^2$$

$$\mathbf{PW_{levo}} = 0.11 - 0.018\ ACN - 0.0038\ TEA + 0.0032\ NaH_2PO_4 + 0.018\ pH + 0.022\ ACN{\times}TEA - 0.0102\ ACN{\times}NaH_2PO_4 - 0.0159\ ACN{\times}pH - 0.0099\ TEA{\times}pH + 0.096\ ACN^2$$

PART C –The RSM diagrams for the six- and four-factor BBD

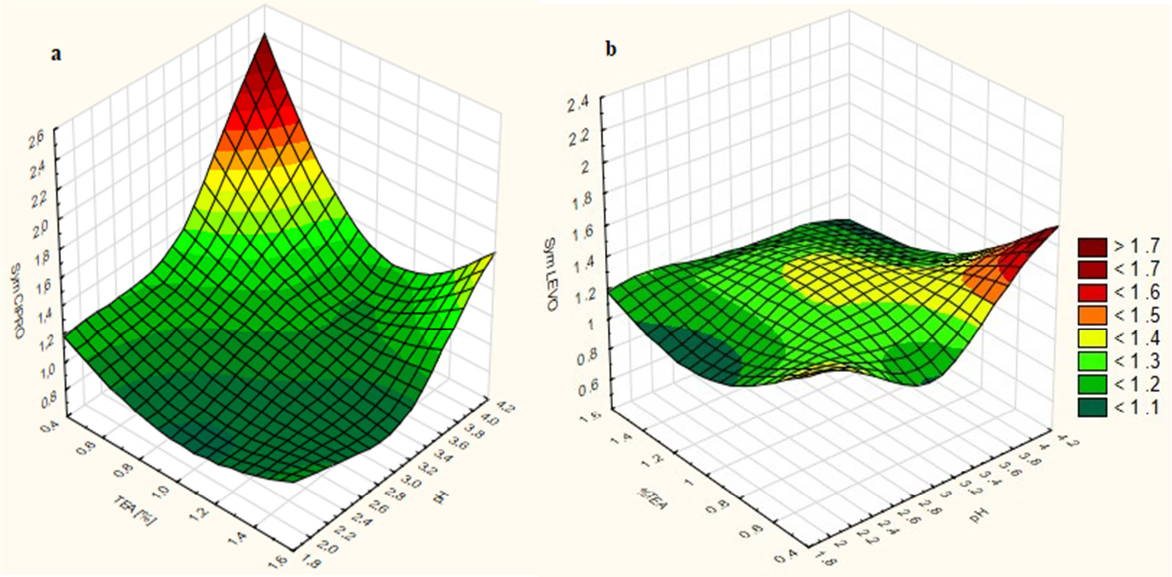

Fig. S.1. The RSM diagram for symmetry (Sym) versus: a) pH and TEA for CIPRO, b) pH and TEA for LEVO for six factors analysis.

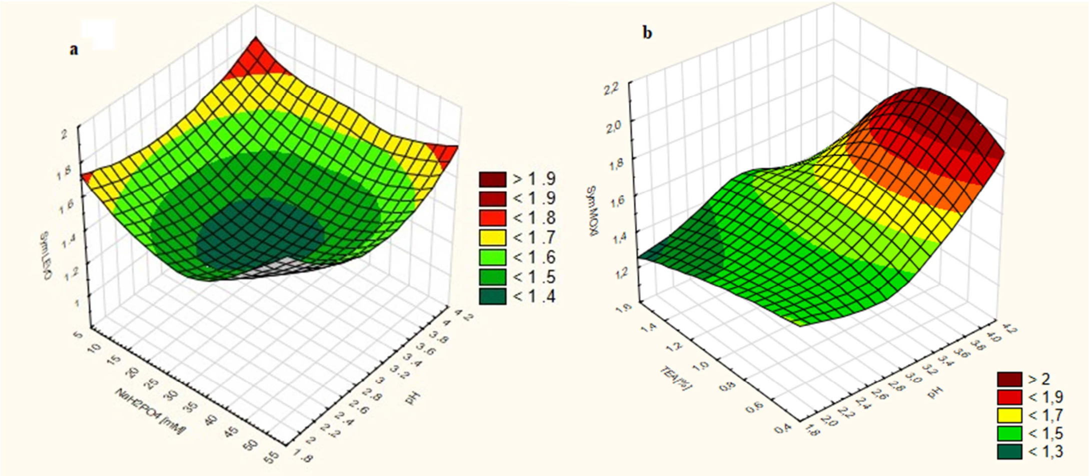

Fig. S.2. The RSM diagram for symmetry (Sym) versus: a) pH and NaH_2PO_4 for LEVO, b) pH and TEA for MOXI for four factors analysis.

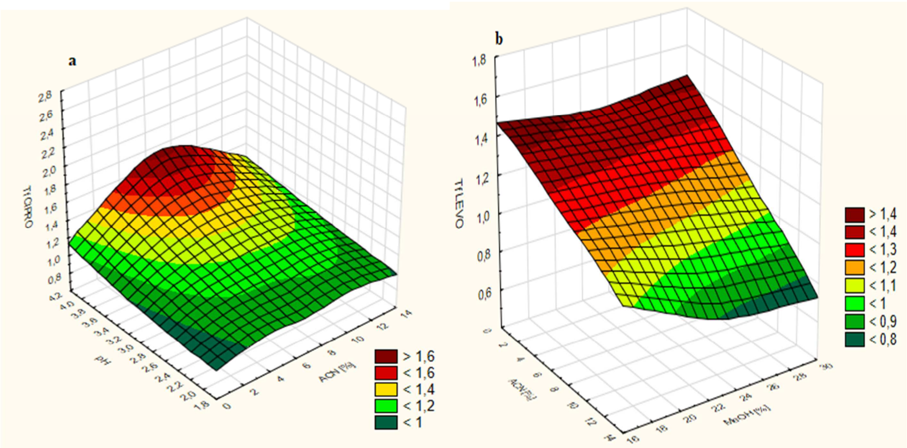

Fig. S.3. The RSM diagram for tailing factor (Tf) versus: a) ACN and pH for CIPRO, b) MeOH and ACN for LEVO for six factors analysis.

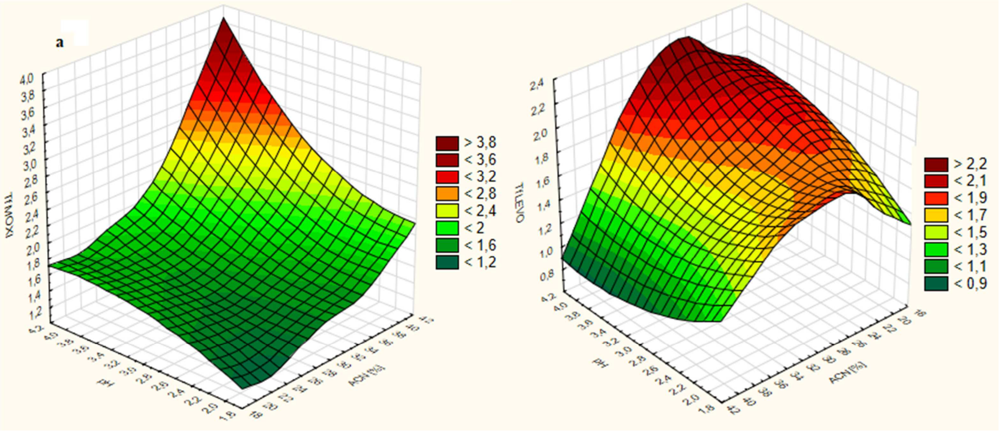

Fig. S.4. The RSM diagram for tailing factor (Tf) versus: a) ACN and pH for MOXI. b) ACN and pH for LEVO for four factors analysis.

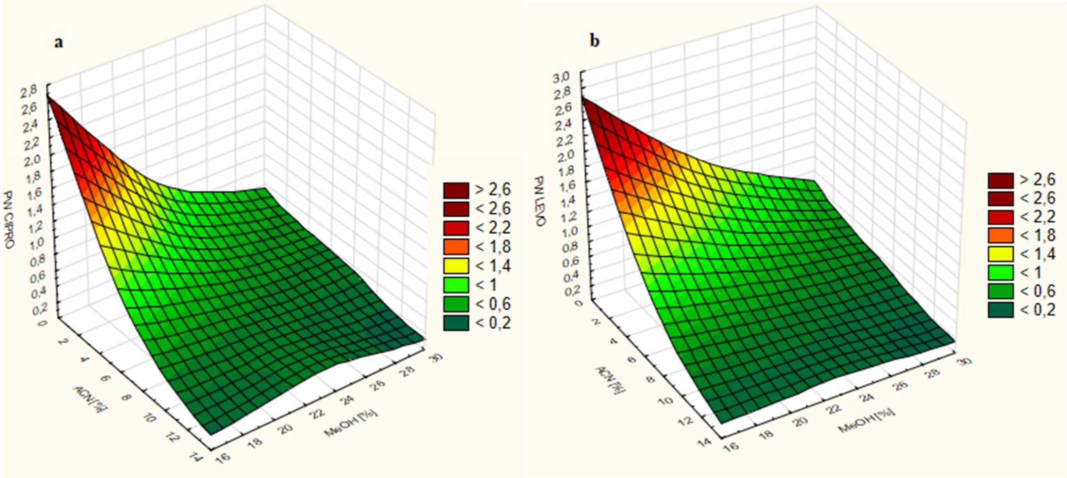

Fig. S.5. The RSM diagram for peak width in half height (PW) versus: a) ACN and MeOH for CIPRO, b) ACN and MeOH for LEVO for six factors analysis.

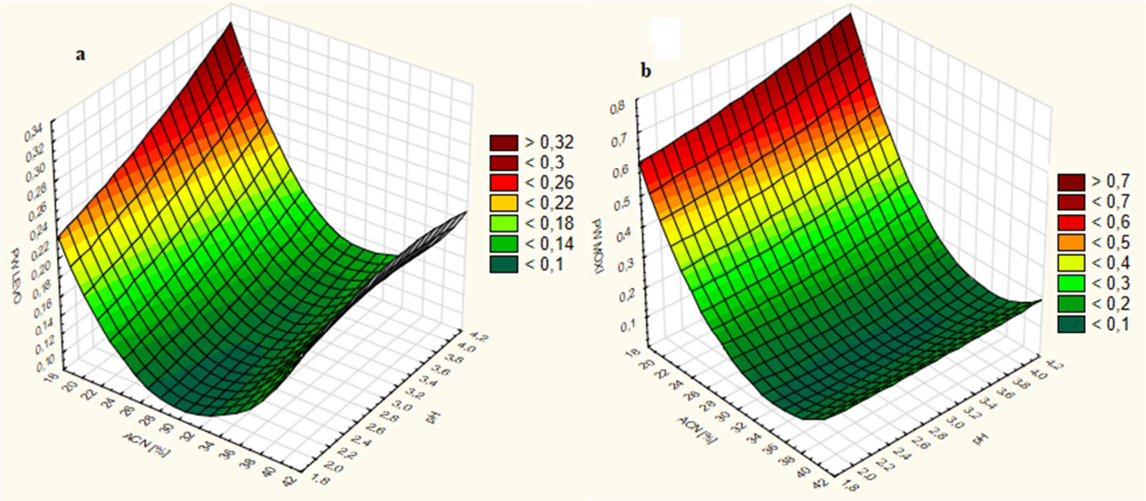

Fig. S.6. The RSM diagram for peak width in half height (PW) versus: a) pH and ACN for LEVO, b) pH and ACN for MOXI for four factors analysis.

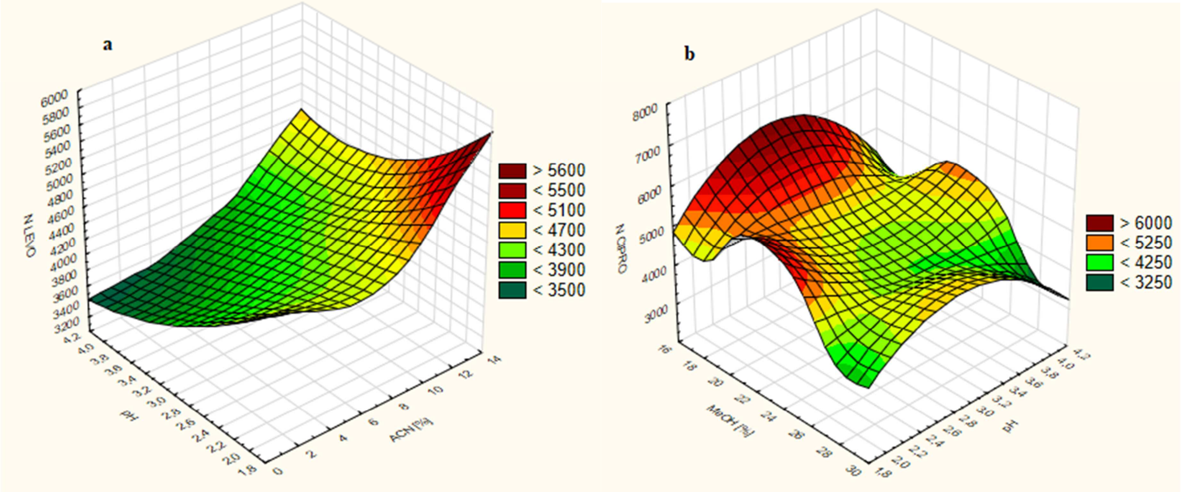

Fig. S.7. The RSM diagram for the number of theoretical plates (N) versus: a) ACN and pH for LEVO, b) MeOH and pH for CIPRO for six factors analysis.

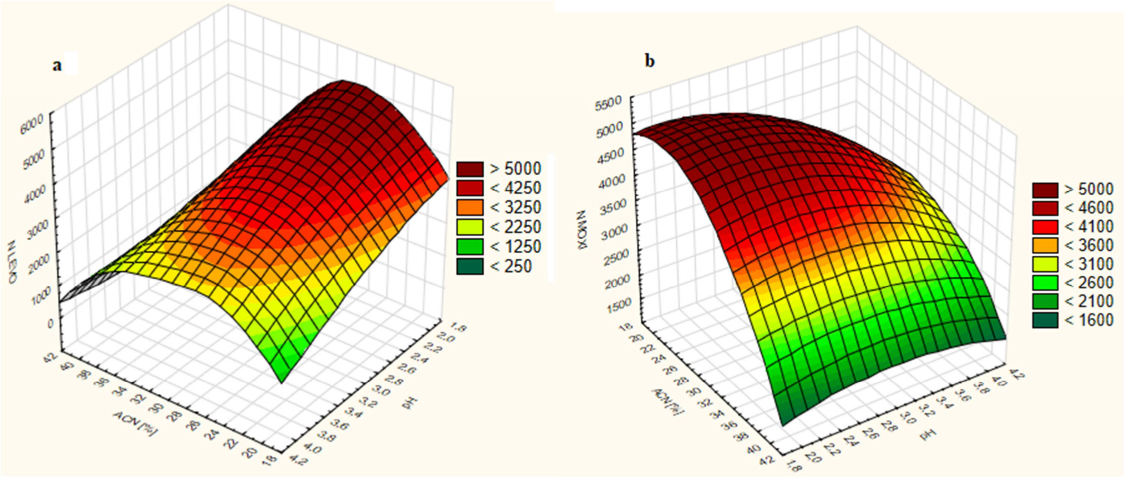

Fig. S.8. The RSM diagram for number of theoretical plates (N) versus: a) pH and ACN for LEVO, b) pH and ACN for MOXI for four factors analysis.

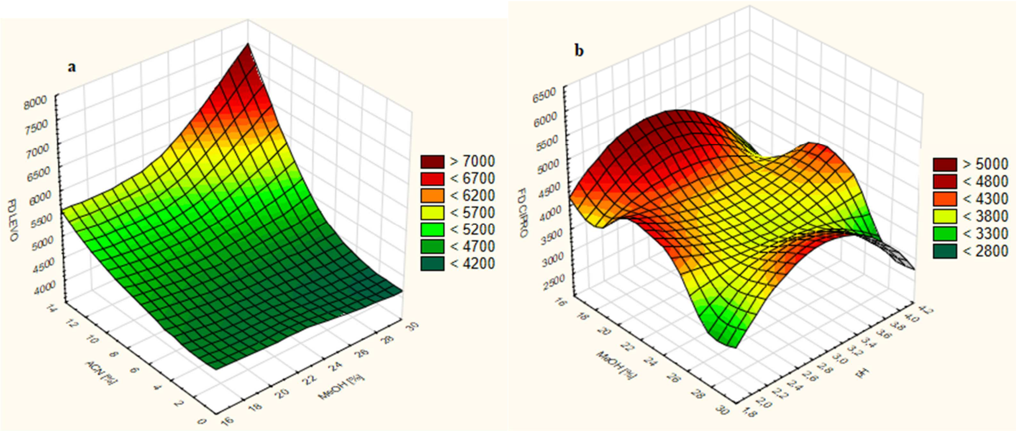

Fig. S.9. The RSM diagram for Foley-Dorsey (FD) versus: a) ACN and MeOH for LEVO, b) MeOH and pH for CIPRO for six factors analysis.

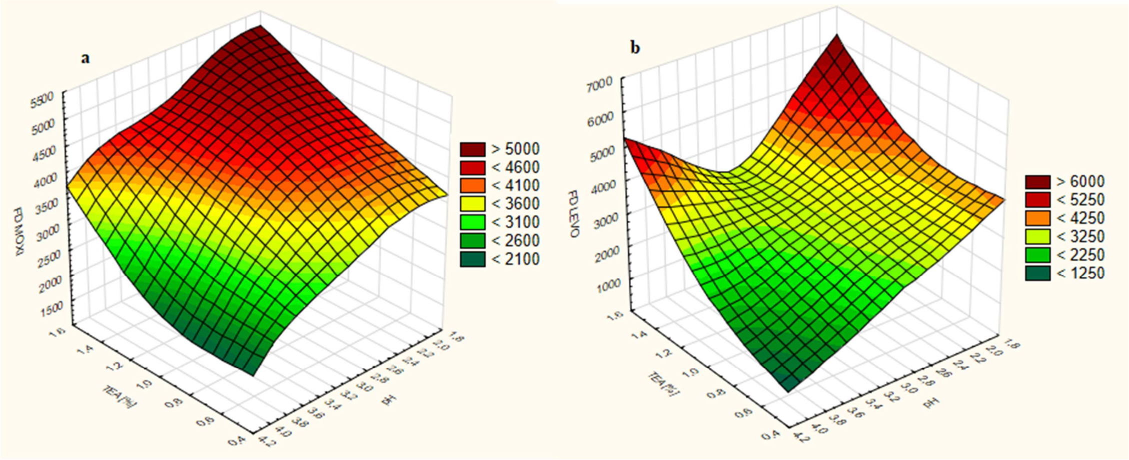

Fig. S.10. The RSM diagram for Foley-Dorsey (FD) versus: a) TEA and pH for MOXI, b) TEA and pH for LEVO for four factors analysis.

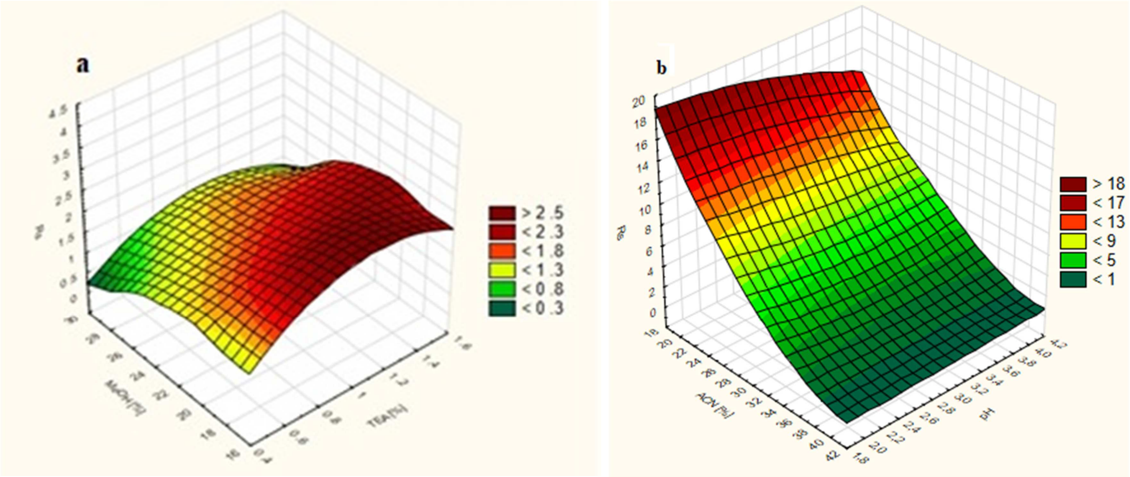

Fig. S.11. The RSM diagram for R_s for: a) MeOH and TEA for CIPRO/LEVO, b) ACN and pH for LEVO/MOXI for four factors analysis.
